# Epidemiological and viral characteristics of undiagnosed HIV infections in Botswana

**DOI:** 10.1186/s12879-022-07698-4

**Published:** 2022-08-28

**Authors:** Lynnette Bhebhe, Sikhulile Moyo, Simani Gaseitsiwe, Molly Pretorius-Holme, Etienne K. Yankinda, Kutlo Manyake, Coulson Kgathi, Mompati Mmalane, Refeletswe Lebelonyane, Tendani Gaolathe, Pamela Bachanas, Faith Ussery, Mpho Letebele, Joseph Makhema, Kathleen E. Wirth, Shahin Lockman, Max Essex, Vlad Novitsky, Manon Ragonnet-Cronin

**Affiliations:** 1 Botswana-Harvard AIDS Institute Partnership, Gaborone, Botswana; 2 Department of Immunology and Infectious Diseases, Harvard T.H. Chan School of Public Health, Boston, MA USA; 3 Ministry of Health and Wellness, Gaborone, Botswana; 4 Centers for Disease Control and Prevention, Atlanta, GA USA; 5 Centers for Disease Control and Prevention, Gaborone, Botswana; 6 Department of Medicine, Division of Infectious Diseases Brigham and Women’s Hospital, Boston, MA USA; 7 Brown University, Providence, RI USA; 8 MRC Centre for Global Infectious Disease Analysis, School of Public Health, Imperial College London, London, UK; 9 Present Address: Department of Ecology and Evolution, University of Chicago, Chicago, USA

**Keywords:** HIV, Undiagnosed infection, Phylogenetics, Recent HIV infection

## Abstract

**Background:**

HIV-1 is endemic in Botswana. The country’s primary challenge is identifying people living with HIV who are unaware of their status. We evaluated factors associated with undiagnosed HIV infection using HIV-1 phylogenetic, behavioural, and demographic data.

**Methods:**

As part of the Botswana Combination Prevention Project, 20% of households in 30 villages were tested for HIV and followed from 2013 to 2018. A total of 12,610 participants were enrolled, 3596 tested HIV-positive at enrolment, and 147 participants acquired HIV during the trial. Extensive socio-demographic and behavioural data were collected from participants and next-generation sequences were generated for HIV-positive cases. We compared three groups of participants: (1) those previously known to be HIV-positive at enrolment (n = 2995); (2) those newly diagnosed at enrolment (n = 601) and (3) those who tested HIV-negative at enrolment but tested HIV-positive during follow-up (n = 147). We searched for differences in demographic and behavioural factors between known and newly diagnosed group using logistic regression. We also compared the topology of each group in HIV-1 phylogenies and used a genetic diversity-based algorithm to classify infections as recent (< 1 year) or chronic (≥ 1 year).

**Results:**

Being male (aOR = 2.23) and younger than 35 years old (aOR = 8.08) was associated with undiagnosed HIV infection (*p* < 0.001), as was inconsistent condom use (aOR = 1.76). Women were more likely to have undiagnosed infections if they were married, educated, and tested frequently. For men, being divorced increased their risk. The genetic diversity-based algorithm classified most incident infections as recent (75.0%), but almost none of known infections (2.0%). The estimated proportion of recent infections among new diagnoses was 37.0% (*p* < 0.001).

**Conclusion:**

Our results indicate that those with undiagnosed infections are likely to be young men and women who do not use condoms consistently. Among women, several factors were predictive: being married, educated, and testing frequently increased risk. Men at risk were more difficult to delineate. A sizeable proportion of undiagnosed infections were recent based on a genetic diversity-based classifier. In the era of “test and treat all”, pre-exposure prophylaxis may be prioritized towards individuals who self-identify or who can be identified using these predictors in order to halt onward transmission in time.

**Supplementary Information:**

The online version contains supplementary material available at 10.1186/s12879-022-07698-4.

## Background

Botswana was among the first countries to reach the Joint United Nations Programme on HIV/AIDS (UNAIDS) 90-90-90 targets, defined as 90% of people living with human immunodeficiency virus (HIV) aware of their status, 90% of those diagnosed on antiretroviral treatment and 90% of those on treatment, virally suppressed [1]. Botswana reached 93-93-98 in 2021 [2]. However, the estimated prevalence of human immunodeficiency virus type 1 (HIV-1) ranges between 18.5 and 21% among adults in the general population and HIV-1 incidence ranges from 0.59 to 1.35% [3–5]

One of the main challenges in curbing transmission is identifying individuals with HIV who are unaware of their HIV status in order to link them to care. Early HIV symptoms are not sufficiently clear or specific to warrant testing, thus there is often a delay between HIV infection and diagnosis. This delayed diagnosis poses an obstacle to the elimination of HIV, as early HIV infection is associated with a disproportionate amount of onward transmission [6–9]. In Sub-Saharan Africa, several factors have been associated with poor HIV testing behaviours and undiagnosed HIV infection [2, 10–16]. These include age, sex, marital status, religion, education, employment status, sexual experience, condom usage [10–12, 17–19]. However, many prior studies have focused on deliberately delineated epidemiological groups such as adolescents, older adults (> 50 years old), or public sector clinic attendees.

To reduce the incidence of HIV-1 in Botswana, it would help to deploy targeted testing strategies for individuals at high risk of being infected or of transmitting the virus. Phylogenetic analysis can provide insights into recent and ongoing transmission events. Thus, it may be possible to utilise phylogenetic analysis to identify high-risk individuals, then describe their demographic or behavioural characteristics. In addition, viral genetic data are informative regarding stage of HIV infection [20–22], so that transmissions in a phylogeny can be interpreted in the context of time since HIV infection.

The Botswana Combination Prevention Project (BCPP) evaluated whether a wide-ranging strategy would reduce HIV incidence over time [3, 4]. 20% of the households across 30 communities were systematically tested for HIV between 2013 to 2018. The study found 601 infections among participants who reported no prior positive test at the start of the study, and 147 individuals seroconverted during follow-up [3]. All study participants completed detailed questionnaires, and HIV-1 full genome sequences were generated from all participants with HIV. Using data from the BCPP study, we sought to evaluate factors associated with undiagnosed HIV-1 infection in Botswana, and to estimate time since infection among new diagnoses.

## Methods

### Study population

The BCPP study enrolled participants from 2013 to 2018 [3]. Approximately 20% of households within 30 communities were randomly selected and eligible participants were enrolled. Each community had an average population size of 5855 for a total trial population of 175,664 [23]. Individuals aged 16 or older completed questionnaires and had blood drawn [3]. We collected questionnaire data in two broad categories: (1) Socio-demographics and community environment: participant and community variables, residency and mobility, education, employment, (2) HIV risk behaviour: HIV testing history and sexual behaviour (see [24] for protocols, questionnaires and data). Negative participants were followed up with yearly HIV tests.

### HIV-1 full genome sequencing

HIV samples from all positively diagnosed participants were sequenced, regardless of antiretroviral therapy (ART) status and viral load. The majority of people with known HIV were already on ART with viral suppression defined as viral load ≤ 400 copies/mL; in these instances, integrated virus was sequenced from viral RNA and proviral DNA templates. Next-generation sequencing (NGS) was performed by the BioPolymers Facility at Harvard Medical School [25] and through collaboration with the PANGEA HIV consortium [26–28] using Illumina platforms MiSeq and HiSeq, as previously described [29–31]. In brief, the nucleic acids were reverse-transcribed and PCR amplified. Amplicons were pooled in equimolar amounts for Illumina library preparation. Sequence assembly was performed de novo using SPAdes version 2.4.0. The HIV-1 reference strain, HXB2 (NC_001802), was used for sequence alignment and a consensus sequence was generated using Abacas version 1.3.1 and MUMmer version 3.2. Next, sequence reads were mapped against the consensus sequence using SMALT version 0.5.0 [31].

HIV consensus sequences were subtyped using COMET [32], and only HIV-1 subtype C sequences were included in our analysis (accounting for > 99% of BCPP sequences). Based on the NGS reads, we were provided with nucleotide frequency files for each patient, detailing the relative frequency of each nucleotide at each site in the alignment. HIV sequences and basic demographic and clinical data are available upon request to the PANGEA consortium [28].

### Statistical analysis

We stratified participants with HIV-1 into three groups as follows: 2995 with previously diagnosed HIV (henceforth referred to as “known cases”), 601 persons with newly diagnosed HIV at enrolment (“new cases”), and 147 persons who seroconverted to HIV-positive during follow-up (“incident cases”). As study participants who tested negative at enrolment were then tested yearly, incident cases were known to have been infected for ≤ 1 year when they were diagnosed. Baseline characteristics and descriptive statistics for all participants have been previously described [3].

We compared responses to the questionnaire across our three groups. We included variables known to be associated with HIV infection and variables known to be associated with undiagnosed infection. We employed logistic regression to compare the characteristics of new cases versus known cases to identify predictors associated with undiagnosed infection. For each variable of interest, we performed univariate analyses comparing the two groups (new cases versus known cases). A single multivariable analysis was fit including demographic and behavioural predictors that were significant (*p* < 0.05) in the univariate analyses. We did not adjust for missing data in the multivariable analysis, and the proportion of complete cases in the data was 53.9%. Demographic predictors evaluated included sex, age, marital status, number of children, religious affiliation, education, and employment status. The behavioural predictors assessed were previous number of HIV tests, sexual activity (yes/no), number of partners, partner concurrency, condom use, condom use frequency in the past year, number of nights spent away from home and partner’s HIV status. Logistic regression was adjusted by community (n = 30) with a random effect using a model with robust standard errors in R. All variables were analysed as categorical variables except for age, which was analysed as a continuous predictor in the primary analysis. A sensitivity analysis was run with age as a categorical variable with five age ranges: 16–24, 25–34, 35–44, 45–54 and 55–64 years old. Finally, we reran the analysis disaggregated by sex, in order to disentangle differential predictors for men and women.

We sought to use viral genetic data to determine whether the time from infection to diagnosis varied significantly between the three groups (new, incident, and known cases). First, we compared the assigned stage of infection based on within-host genetic diversity (< or ≥ 1 year) for each group, using a χ^2^ test. We then compared the recency probability distributions between the three groups using a Kruskal–Wallis test. These comparisons were conducted for participants for whom deep sequencing nucleotide frequency data were available (n = 1867). Finally, we compared the length of terminal branch lengths across the groups, and across each HIV-1 gene (*gag, pol, env*), in a pairwise manner, using Kolmogorov–Smirnov (KS) tests. These comparisons were conducted for participants for whom consensus genetic sequences were available for at least one gene (n = 2872). This dataset included 2339 known cases, 399 new cases and 134 incident cases. To account for multiple non-independent comparisons (across different genes), we used Bonferroni’s correction to assess statistical significance where appropriate. All statistical analyses were conducted in R (version 3.6.0) [33].

### Phylogenetic analysis

For participants for whom viral genetic data were available (n = 2872), we constructed phylogenies separately for each HIV gene region: *gag*, *pol* and *env*. Sequences were available for 2339 known cases, 399 new cases and 134 incident cases. We compared the viral characteristics between those two groups (know vs. new cases), and a third: those diagnosed with incident infections during the BCPP trial (n = 147). Because this latter group were negative at the start of the trial, then tested yearly, we knew their infections were < 1 year when they were diagnosed. We wanted to use our two reference groups (known older cases, and incident cases) to evaluate how long those diagnosed at the start of trial were likely to have been infected before they were diagnosed.

Maximum likelihood phylogenies were reconstructed using RaxML [34] under a GTR model with four gamma rates. Phylogenies included sequences from an additional 3277 patients from Botswana clinics, to serve as local controls. Final phylogeny sizes were: *gag* (n = 5631), *pol* (n = 6084) and *env* (n = 5840). We time-resolved the phylogenies using the treedater package, available in R [35], using sample times as tip dates. For each tip, we extracted terminal branch lengths (measured in time) using the “Analyses of Phylogenetics and Evolution” (APE) [36] and phytools [37] R packages. We compared the distributions of terminal branch lengths across our three groups (known cases, new cases, incident cases) using Kolmogorov–Smirnov tests.

### Inference of stage of HIV infection

For each study participant for whom deep sequencing nucleotide frequency data were available (n = 1867), we calculated the probability (0–1) of their infection being recent (< 1 year) based on within-host genetic diversity, demographic (age, sex) and clinical (treatment status, viral load) predictors using an xgboost gradient boosting [38] machine learning algorithm. The machine learning classifier is trained on a dataset of known recent (< 1 year) and chronic (≥ 1 year) infections to classify stage of HIV infection for individuals in each of our three groups. This analysis was conducted on all individuals for whom NGS coverage was sufficient to derive site-specific nucleotide frequency distributions (n = 1867). We selected the threshold that optimized for accuracy (the highest number of overall correct classifications). The algorithm has been previously developed for and trained on the BCPP dataset on participants with known duration of infection [39]. For each participant in the present study, we used the algorithm to predict the probability of recency. We carefully excluded individuals comprised in the present study from the dataset used to train the classifier.

## Results

### Participant demographics

In total, 12,610 people were enrolled in BCPP. Of these, 8050 (63.8%) were women and 4560 (36.2%) were men. A total of 3596 (29%) participants tested positive for HIV-1 at enrolment and 147 participants seroconverted during the study.

#### Predictors associated with undiagnosed HIV infection at baseline

##### Demographic predictors of undiagnosed HIV infection at baseline

We compared new cases to known cases to identify factors associated with undiagnosed infection. The analysis was conducted on the group as a whole and repeated on women and men separately, demonstrating stark differences by sex. Men were more likely than women to have an undiagnosed HIV infection (Additional file 1: Table S1). As participants of both sexes aged, they were less likely to have an undiagnosed HIV infection (aOR = 0.94 per year, *p* < 0.001; Additional file 1: Table S1). But for women, only those aged 16–24 had an increased chance of an undiagnosed infection (aOR = 4.63, *p* < 0.001), while for men age groups 16–24, 25–34, 35–44 were all more likely to harbour undiagnosed infections than the reference group (55–64; Table 1). For women, being single (aOR = 0.73, *p* < 0.001) and divorced or widowed (aOR = 0.47, *p* < 0.001) significantly reduced the odds of having an undiagnosed infection as compared to being married. This effect was inversed for men, where being single (aOR = 1.72, *p* < 0.001) or divorced (aOR = 3.95, *p* < 0.05) increased the odds of having an undiagnosed infection as compared to being married. Next, not having children increased the odds of a woman harbouring an undiagnosed infection compared to having children (OR = 0.53, *p* < 0.001), and the more children a woman had the less likely she was to have an undiagnosed infection. However, due to collinearity with sex (the question was asked only to women), the effect of child number could not be evaluated in men. Women who were not affiliated with any religion were more likely to have undiagnosed HIV infections (aOR = 1.33, *p* < 0.05), but there was no such effect in men. For women, the odds of an undiagnosed infection increased slightly with attaining higher education beyond Senior Secondary level (*p* < 0.05), but this was not the case for men.

**Table 1:** Logistic regression for demographic characteristics associated with undiagnosed infection by sex

Variable	Category	Women (n = 383 newly diagnosed, n = 2252 known cases)		Men (n = 218 newly diagnosed, n = 743 known cases)	
		OR (95% CI)^a^	aOR (95% CI)^b^	OR (95% CI)	aOR (95% CI)
Age	16–24 years	5.42*** (4.29–6.89)	4.63*** (2.63–8.18)	4.58*** (2.73–7.98)	12.7** (1.32–121.7)
	25–34 years	0.99 (0.83–1.17)	0.81 (0.51–1.28)	4.57*** (3.25–6.43)	5.68*** (2.54–12.7)
	35–44 years	0.29*** (0.25–0.35)	0.36*** (0.22–0.57)	2.64*** (1.94–3.59)	3.16** (1.45–6.87)
	45–54 years	0.54*** (0.45–0.65)	0.95 (0.66–1.39)	0.98 (0.7–1.35)	1.0 (0.48–2.14)
	55–64 years	*Ref*		*Ref*	
Marital status	Married	*Ref*		*Ref*	
	Single/Never married	0.73*** (0.63–0.84)	0.45*** (0.33–0.6)	2.58*** (2.0–3.27)	1.72 (1.0–2.05)
	Divorced/Widowed	0.47*** (0.38–0.58)	0.25*** (0.16–0.39)	1.31 (0.79–2.19)	3.95^*^ (1.3–11.98)
Number of children^c^	None	*Ref*			
	1–5 children	0.53**(0.33–0.87)	–	–	–
	≥ 5 children	0.36***(0.21–0.64)	–	–	–
Religious affiliation	Affiliated with religion	*Ref*		*Ref*	
	No religious affiliation	1.1 (0.98–1.25)	1.33^*^ (1.06–1.67)	0.85 (0.7–1.04)	0.8 (0.57–1.11)
Education level	Tertiary	*Ref*		*Ref*	
	Senior secondary	0.93 (0.73–1.18)	0.64^*^ (0.44–0.92)	1.5 (0.93–2.45)	1.55 (0.75–3.19)
	Junior secondary	0.29*** (0.24–0.35)	0.37*** (0.26–0.53)	1.91*** (1.31–2.75)	1.42 (0.78–2.60)
	Primary	0.21*** (0.17–0.26)	0.38*** (0.23–0.61)	0.71 (0.49–1.04)	1.19 (0.61–2.29)
	Non-formal	0.27*** (0.22–0.34)	0.44** (0.26–0.75)	0.53*** (0.35–0.79)	0.94 (0.4–2.2)

*n* number of participants, *OR* odds ratio, *aOR* adjusted odds ratio for clustering by community, *CI* confidence interval, *Ref* reference group

^*^p < 0.05, **p < 0.01, ***p < 0.001

^a^Univariate logistic regression

^b^Multivariable logistic regression

^c^Excluded from the multivariable due to interaction with other variables

##### Behavioural predictors of undiagnosed HIV infection at baseline

Women who tested for HIV more frequently were more likely to have undiagnosed infections (Table 2, aOR = 18.88 for those with ten or more tests compared to those with 1 or 2, *p* < 0.001) but no such effect was observed in men. In the multivariable model, number of sexual partners and concurrent partners were not significantly predictive (*p* > 0.05). Women who reported knowing their partner’s HIV status to be negative were more likely to have undiagnosed HIV infection than those who reported their partners to be HIV positive (*p* < 0.001). Condom use in the past year was a significant predictor of undiagnosed infection among women in the univariate logistic regression (OR = 2.37, *p* < 0.001), but it was excluded from the multivariable analysis due its interaction with condom frequency. Participants who never used condoms, and those who used them only sometimes, were more likely to have undiagnosed infection than those who always used them (*p* < 0.001 for women and *p* < 0.05 for men). Increased time away from home increased the risk of undiagnosed infection in the univariate analysis for women (OR = 1.24 and 1.54, *p* < 0.001) but not in the multivariable analysis.

**Table 2:** Logistic regression for behavioural characteristics associated with undiagnosed infection by sex

Variable	Category	Women (n = 383 newly diagnosed, n = 2252 known cases)		Men (n = 218 newly diagnosed, n = 743 known cases)	
		OR (95% CI)^a^	aOR (95% CI)^b^	OR (95% CI)	aOR (95% CI)
Number of HIV tests	1–2	*Ref*		*Ref*	
	3–4	3.74*** (3.28–4.27)	4.81*** (3.72–6.23)	0.76 (0.58–1.0)	0.74 (0.49–1.1)
	4–9	6.41*** (5.43–7.61)	10.9*** (7.82– 15.2)	0.73 (0.51–1.07)	0.49^*^ (0.28–0.85)
	10 +	9.8*** (6.77–14.74)	18.8*** (8.41–42.1)	1.1 (0.48–3.03)	1.0 (0.36–3.0)
Number partners in 1 year	1 partner	*Ref*		*Ref*	
	2 partners	1.15 (1.0–1.32)	0.88 (0.15–5.1)	1.32*** (1.0–1.68)	0.67 (0.02– 20.0)
	3 partners	2.54*** (1.43–4.93)	4.6 (0.25– 84.6)	1.17 (0.5–2.68)	0.19 (0.004–9.8)
	4 partners	1.34 (0.66–3.0)	0.52 (0.09–3.0)	1.87 (0.73–4.61)	1.0 (0.03–35.1)
	None	0.71*** (0.62–0.82)	–	1.36 (1.0–1.86)	–
Concurrency in past year	No concurrent partners	*Ref*		*Ref*	
	Concurrent partners	1.17^*^ (1.0–1.34)	1.2 (0.21–6.9)	1.42*** (1.12–1.79)	1.68 (0.06–46.8)
Partner’s HIV status	HIV negative	*Ref*		*Ref*	
	HIV positive	0.06*** (0.05–0.07)	0.09*** (0.07–0.12)	0.8 (0.6–1.07)	0.8 (0.6–1.06)
Condom use in past year	No	*Ref*		*Ref*	
	Yes	2.37*** (2.11–2.66)	–	0.8 (0.65–1.0)	–
Condom frequency	Always	*Ref*		*Ref*	
	Sometimes	1.98*** (1.75–2.25)	1.64*** (1.32–2.03)	0.83 (0.66–1.05)	1.56^*^ (1.07–2.25)
	Never	3.07*** (2.53–3.75)	2.9*** (2.15–3.89)	0.77 (0.53–1.14)	2.17** (1.25–3.76)
Nights away from home	Zero	*Ref*		*Ref*	
	< 1 week–< 1 month	1.24*** (1.09–1.42)	0.88 (0.67–1.15)	0.81 (0.64–1.03)	1.0 (0.65–1.68)
	> 1 month	1.54*** (1.38–1.73)	0.96 (0.77–1.20)	0.85 (0.69–1.06)	0.95 (0.56–1.59)

*n* number of participants, *OR* odds ratio, *aOR* adjusted odds ratio for clustering by community, *CI* confidence interval, *Ref* reference group

^*^p < 0.05, **p < 0.01, ***p < 0.001

^a^Univariate logistic regression

^b^Multivariable logistic regression

### Differences in assigned timing of HIV infection

**Fig. 1:**
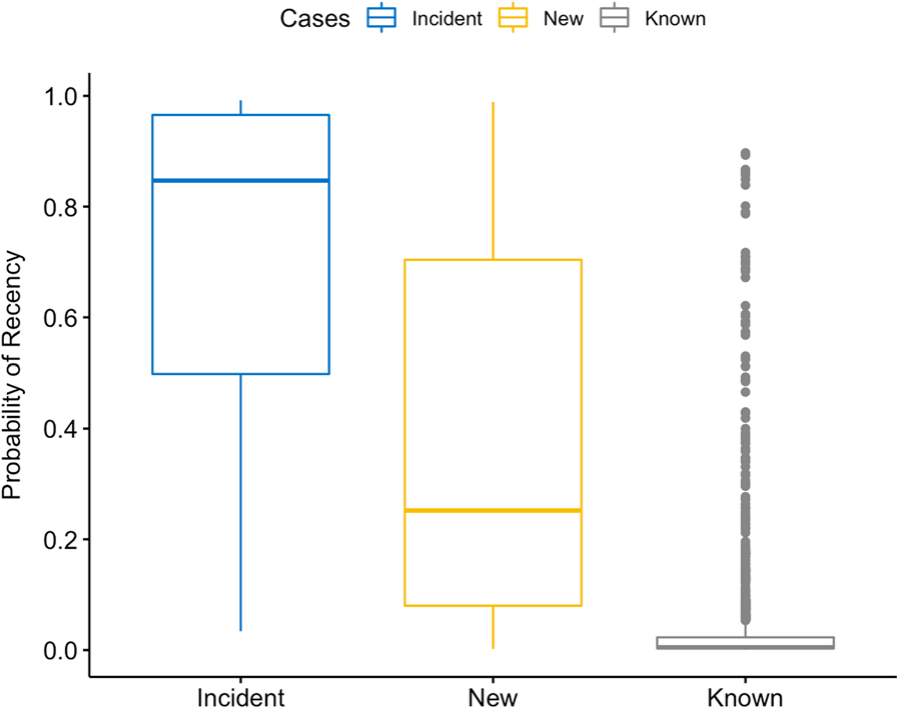
Recency prediction based on diversity-based classification algorithm. The distribution of data is skewed around the median, shown by the middle line. Boxes represent quartiles (25%, 75%) and whiskers represent the range of the data. Dots represent outliers in the plot. The Kruskal–Wallis test was used to calculate the *p*-value (*p* < 10^–16^). Incident cases mean probability of recency was 0.847, for new cases it was 0.252 and for known cases it was 0.005

Results were aligned with those expected: incident cases were nearly all classified as recent infections (75%), whereas 37% of newly diagnosed cases and only 2% of known cases were classified as recent (Table 3, p < 0.001 based on the Pearson χ^2^ test). The classifier produces a probability of recency for each individual, rather than assigning a categorical stage, and we observed the same pattern when we compared the probability distributions across the three groups (Fig. 1). Incident cases had the highest probability of recency (p = 0.847) while known cases had the lowest probability (p = 0.005). New cases had an intermediary probability of being recently infected (p = 0.252).

**Table 3:** Timing of HIV infection among all participants

Variables	Incident cases N = 136^#^	New cases N = 284^#^	Known cases N = 1447^#^	*p*-value
Recent, n (%)	102 (75.0%)	105 (37.0%)	29 (2.0%)	
Chronic, n (%)	34 (25.0%)	179 (63.0%)	1418 (98.0%)	**< 0.001**

^#^Analysis for participants with available data, n—number of participants. Non-independence between groups was evaluated using Pearson χ^2^ test for categorical data

### Differences in terminal branch lengths

The statistical analysis for terminal branch lengths across three groups and three genes amounted to nine non-independent comparisons, therefore we set our threshold for significance to p < 0.0055. We observed no consistent pattern of difference in terminal branch lengths between groups.

## Discussion

Among a large representative population of adults with HIV in rural/peri-urban Botswana, we compared the demographic and behavioural characteristics of those with known, diagnosed HIV-infection compared with those newly diagnosed with HIV at the start of a clinical trial. Participants with undiagnosed HIV infections were more likely to be male, young, and not to consistently use condoms. Among women, being married, educated and testing frequently increased the risk of an undiagnosed HIV infection. For men, there was a wider range of ages among undiagnosed HIV cases and being divorced increased risk.

Viral genetic sequences are informative regarding stage of HIV infection because individuals are usually infected with a single virus and genetic diversity then increases with time [20–22]. Results from the genetic diversity-based classifier were concordant with epidemiological data: most known cases were classified as chronic (97.9%) while most incident cases were classified as recent (75.0%). This means that 25% of incident infections were misclassified as chronic, a high false negative rate due to a high proportion of participants being on ART [39]. Based on the classifier, over a third (37.0%) of newly diagnosed cases were recently infected. Given the high false negative rate of the classifier this proportion may be an underestimate—therefore a sizeable proportion of newly diagnosed cases were likely to be recent infections. Another genetic signature of time between infection and diagnosis is the length of the terminal branch leading to a sequence [22, 40, 41]. Short terminal branch lengths indicate that sampling occurred shortly after the last transmission event. However, we found no consistent differences in the distribution of terminal branch lengths among our three groups. This lack of signal may be due to the low sample proportion of our population as a whole (12,610 of a total trial area population of 175,664, 7.2%): the relationship between terminal branch length and time to diagnosis will be disrupted if too many transmissions are missed in the phylogeny [42]. The relationship between time to diagnosis and terminal branch length has been demonstrated in simulations [41] but its utility in the real world will have to be further ascertained in datasets with higher sampling proportions. One study that successfully used root to tip branch length in determining time since infection demonstrated its use for estimating HIV incidence at a population level [43].

In our study, more women (63.8%) were enrolled than men (36.2%). A similar study in Zambia and South Africa (HPTN-071) showed similar enrolment patterns for women (70%) and men (30%) [44]. Surveys across sub-Saharan Africa have consistently demonstrated that HIV testing uptake is higher among women than among men [2, 13, 45]. One reason women are more likely to know their HIV status is that most countries, including Botswana, screen for HIV during pregnancy as part of prevention of mother to child transmission [3, 46, 47]. This strategy may explain why in our study, women with more children were less likely to have undiagnosed HIV infections. Furthermore, fewer undiagnosed infections were observed as participants increased in age, in agreement with others [2, 13, 19, 45], and testing increased with age. HIV testing rates among men below 25 years are low [17]. In Botswana, people above 50 living with HIV are more likely to be aware of their HIV status and to be on antiretroviral therapy [36]. Older women in particular are more likely to be aware of their positive HIV status than older men [48], and concordantly, in our study the effect of age was much stronger in women than in men. Men up to age 44 had an increased risk for an undiagnosed infection, while this was only true for women up to age 24.

Our data shows that being married increased the risk of an undiagnosed HIV infection for women. The association between marriage and undiagnosed HIV infection has been noted previously [10, 11, 49]. In contrast, a survey conducted in South Africa showed that married people living with their spouses were *less* likely to be HIV positive [50], but even in that study, HIV infections were highest among those who were married but who spent extended periods away from home. Knowing one’s partner to be HIV-positive decreased the odds of an undiagnosed HIV infection. This finding could indicate that sero-discordant couples take precautions to prevent transmission and to get diagnosed rapidly but it seems reasonable to assume that this is most likely due to demonstrate the success of treatment as prevention. In our study, we found that those women with a higher education were more likely to have undiagnosed HIV infections, despite education improving HIV testing behaviours [10, 46, 49]. Previous cross-sectional analyses have demonstrated a positive association between educational attainment and HIV positivity across sub-Saharan African countries [51]; educational attainment may increase the likelihood of sexual opportunities or risk-taking.

The behavioural factors associated with undiagnosed HIV were repeated HIV testing (for women) and inconsistent condom use (for both sexes). At first glance, this first finding seems contradictory: frequent testing increases the chances of diagnosing HIV within the early stages of infection and should decrease the probability that a person has an undiagnosed HIV infection. Most participants (72.8% of known, previously diagnosed cases and 44.6% newly diagnosed cases) were tested for the first-time at enrolment or had tested only once previously. It is possible that women who test frequently do so because they are aware of being at risk for HIV. For example, they may use HIV testing as a greenlight for unprotected sex, or they may get tested following a risk event. However, higher numbers of partners and concurrency were not identified among these women. They may have risk behaviours that they did not disclose within this study. Further research is required to make specific recommendations regarding optimal HIV testing timings and frequency for this group, who already test regularly. We note that once a person tests HIV positive, they will not continue to get tests. Therefore, there is some censoring in our data on number of tests, however this censoring should not bias our interpretation. Health seeking behaviours such as frequent HIV testing and safe sexual practices by using a condom reduce HIV transmission [12]. Consistent condom use offered protection against HIV infection and participants who never used condoms were 4 times more likely to have undiagnosed HIV than those who always did.

Our analysis was subject to several limitations. First, the BCPP study enrolled only 20% of households in target areas. While this proportion is high for a single study, it is low when compared to HIV testing and sequencing coverage in countries such as the UK. A similar study, PopART, conducted in Zambia and South Africa sampled 4.8% of their population (48,301/100,000) [44] while BCPP reached 7.2% (12,610/175,664). It may be due to this low sampling proportion that we were not able to see differences in terminal branch lengths, because too many transmissions were missed in the phylogeny. Lastly, although the BCPP questionnaires were thorough, many answers were not complete. Our analyses were affected by missing data in some variables of interest ranging from 1–40%. Our analysis yielded significant results, but if some information was deliberately obscured, we may be missing important associations. Further studies are warranted to further investigate these variables with complete datasets.

## Conclusion

Despite the tremendous success of the “Test and Treat all” strategy in Botswana, 16.7% (601/3596) of BCPP participants with HIV at enrolment did not know their positive status. Taken together, our results indicate that adults with undiagnosed infections are likely to be young (especially women), and to not consistently use condoms with their partners. Women were more likely to have undiagnosed infections if they were married, educated, and tested frequently. Men are more likely to have undiagnosed HIV infections and being divorced increased their risk but otherwise they did not stand out in obvious ways from men with diagnosed infections. Notably, a sizeable proportion of undiagnosed infections were likely to be recent based on a genetic-diversity-based classifier, suggesting they are aware of their risk. Our results stress the importance of targeting interventions towards men in a range of places where they might be useful; for example, by offering HIV self-testing or testing in workplaces, sporting events, barber shops or places where men congregate regularly. Clearer recommendations may be needed as to how frequently, and under what circumstances, this group should get tested for HIV. With high rates and coverage of HIV testing and antiretroviral therapy initiation, incorporating the identified predictors to prioritize HIV testing and pre-exposure prophylaxis (PreP) will help reduce national HIV incidence in Botswana.

## Supplementary Information

**Additional file 1: Table S1** Demographic and behavioural factors associated with newly diagnosed HIV-1 infections (n = 601) compared to known HIV-cases (n = 2995) in Botswana. **Figure S1.** Differences in terminal branch lengths for three gene regions: A. Gag, B. Polymerase and C. Envelope genes of the HIV-1C virus. Incident cases (blue), new cases (yellow) and known cases (grey) are shown in each plot. The Student’s t test was used to generate the *p*-values: ns -not significant, *p < 0.05, **p < 0.01, ***p < 0.001. After Bonferroni’s correction, no statistical difference was observed in terminal branch lengths across the HIV genes.

## Data Availability

Sequence data and basic demographics for this study are available upon request to the PANGEA HIV consortium (www.pangea-hiv.org). BCPP protocols and collected data are made available at https://data.cdc.gov/Global-Health/Botswana-Combination-Prevention-Project-BCPP-Publi/qcw5-4m9q. For full access, please use the data request form.

## Abbreviations

HIV: Human immunodeficiency virus
BCPP: Botswana Combination Prevention Project
NGS: Next generation sequencing
KS: Kolmogorov–Smirnov
n: Number of participants
OR: Odds ratio
aOR: Adjusted odds ratio
CI: Confidence interval
Ref: Reference group
