# Supplementary material for: Epidemiological and viral characteristics of undiagnosed HIV infections in Botswana
Source: BMC Infect Dis. 2022 Aug 28;22:710. doi: 10.1186/s12879-022-07698-4 (PMC9420270; doi:10.1186/s12879-022-07698-4)
Supplement: Supplementary file 1 — Additional file 1: Table S1 Demographic and behavioural factors associated with newly diagnosed HIV-1 infections (n = 601) compared to known HIV-cases (n = 2995) in Botswana. Figure S1. Differences in terminal branch lengths for three gene regions: A. Gag, B. Polymerase and C. Envelope genes of the HIV-1C virus. Incident cases (blue), new cases (yellow) and known cases (grey) are shown in each plot. The Student’s t test was used to generate the p-values: ns -not significant, *p < 0.05, **p < 0.01, ***p < 0.001. After Bonferroni’s correction, no statistical difference was observed in terminal branch lengths across the HIV genes. [file 12879_2022_7698_MOESM1_ESM.docx]

Epidemiological and viral characteristics of undiagnosed HIV infections in Botswana

Lynnette Bhebhe^1^, Sikhulile Moyo^1,2^, Simani Gaseitsiwe^1,2^, Molly Pretorius-Holme^2^, Etienne K. Yankinda^1^, Kutlo Manyake^1^, Coulson Kgathi^1^, Mompati Mmalane^1^, Refeletswe Lebelonyane^3^, Tendani Gaolathe^1^, Pamela Bachanas^4^, Faith Ussery^4^, Mpho Letebele^5^, Joseph Makhema^1,2^, Kathleen E. Wirth^4,5^, Shahin Lockman^1,2,6^, Max Essex^2^, Vlad Novitsky^2,7^, Manon Ragonnet-Cronin^8*^

^1^Botswana-Harvard AIDS Institute Partnership, Gaborone, Botswana; ^2^Department of Immunology and Infectious Diseases, Harvard T.H. Chan School of Public Health, Boston, MA, USA; ^3^Ministry of Health and Wellness, Gaborone, Botswana, ^4^Centers for Disease Control and Prevention, Atlanta, GA, USA; ^5^Centers for Disease Control and Prevention, Gaborone, Botswana; ^6^Department of Medicine, Division of Infectious Diseases Brigham and Women’s Hospital, Boston, MA, USA; ^7^Brown University, Providence, RI, USA; ^8^MRC Centre for Global Infectious Disease Analysis, School of Public Health, Imperial College London, UK

Additional information

**Table S1** Demographic and behavioural factors associated with newly diagnosed HIV-1 infections (n=601) compared to known HIV-cases (n=2,995) in Botswana

| **Variable** | **Category** | **Known cases (%)** | **New cases (%)** | **OR (95% CI)*^a^*** | **aOR (95% CI)*^b^*** |
| --- | --- | --- | --- | --- | --- |
| **Sex** | **Female** | 2252 (75.2%) | 383 (63.7%) | *Ref* |  |
|  | **Male** | 743 (24.8%) | 218 (36.3%) | 1.85^***^(1.48 – 2.3) | 2.23^***^ (1.84 – 2.7) |
| **Age (years)** | **Median (IQR)** | 40.8 (34.5–49.1) | 34.7(28.4–43.6­­) | 0.95^***^ (0.94 – 0.96) | 0.94^***^ (0.93– 0.95) |
| **Marital status** | **Married** | 478 (16%) | 62 (10.3%) | *Ref* |  |
|  | **Single/Never married** | 2288 (76.4%) | 511 (85%) | 0.99(0.89 – 1.11) | 0.46^***^ (0.38 – 0.58) |
|  | **Divorced/Widowed** | 227 (7.6%) | 27 (4.5%) | 0.5^***^(0.41 – 0.6) | 0.49^***^ (0.36 – 0.67) |
|  | **Missing** | 2 (0.1%) | 1 (0.2%) |  |  |
| **Religious affiliation** | **Affiliated with religion** | 2166 (72.3%) | 381 (63.4%) | *Ref* |  |
|  | **No religious affiliation** | 828 (27.6%) | 220 (36.6%) | 1.57^***^(1.26 – 1.96) | 1.14(0.95 – 1.35) |
|  | **Missing** | 1 (0%) | 0 (0%) |  |  |
| **Education level** | **Tertiary** | 189 (6.3%) | 57 (9.5%) | *Ref* |  |
|  | **Senior secondary** | 225 (7.5%) | 86 (14.3%) | 0.99(0.81 – 1.22) | 0.83(0.61 – 1.16) |
|  | **Junior secondary** | 1169 (39%) | 243 (40.4%) | 0.34^***^(0.29 – 0.4) | 0.43^***^(0.32 – 0.58) |
|  | **Primary** | 927 (31%) | 145 (24.1%) | 0.19^***^(0.16 – 022) | 0.55^***^(0.38 – 0.79) |
|  | **Non-formal** | 462 (15.4%) | 68 (11.3%) | 0.22^***^(0.18 – 0.26) | 0.69 (0.48 – 1.0) |
|  | **Missing** | 23 (0.8%) | 2 (0.3%) |  |  |
| **Number of HIV tests^c^** | **1-2** | 2180 (72.8%) | 268 (44.6%) | *Ref* |  |
|  | **3-4** | 474 (15.8%) | 99 (16.5%) | 3.22^***^(2.87 – 3.61) | 3.96^***^(3.10 – 5.07) |
|  | **4-9** | 228 (7.6%) | 61 (10.1%) | 5.41^***^(4.67 – 6.29) | 8.13^***^(5.80 – 11.50) |
|  | **10** | 35 (1.2%) | 14 (2.3%) | 9.26^***^(6.64 – 13.35) | 14.4^***^(6.60 – 31.20) |
|  | **Missing** | 78 (2.6%) | 159 (26.5%) |  |  |
| **Number partners in 1 year** | **1 partner** | 1704 (56.9%) | 327 (54.4%) | *Ref* |  |
|  | **2 partners** | 515 (17.2%) | 131 (21.8%) | 1.31^***^(1.18 – 1.47) | 0.76(0.30– 1.93) |
|  | **3 partners** | 26 (0.9%) | 16 (2.7%) | 3.18^***^(2.15 – 4.90) | 1.73(0.53 – 5.68) |
|  | **4 partners** | 25 (0.8%) | 17 (2.8%) | 2.57^***^(1.72 – 4.01) | 0.81(0.27 – 2.45) |
|  | **None** | 476 (15.9%) | 66 (11%) | 0.74^***^(0.66 – 0.84) | – |
|  | **Missing** | 249 (8.3%) | 44 (7.3%) |  |  |
| **Concurrency in past year** | **No concurrent partners** | 1720 (57.4%) | 332 (55.2%) | *Ref* |  |
|  | **Concurrent partners** | 550 (18.4%) | 159 (26.5%) | 1.42^***^(1.28 – 1.59) | 1.49(0.58 – 3.82) |
|  | **Missing** | 725 (24.2%) | 110 (18.3%) |  |  |
| **Partner’s HIV status^d^** | **HIV negative** | 576 (19.2%) | 161 (26.8%) | *Ref* |  |
|  | **HIV positive** | 1221 (40.8%) | 102 (17%) | 0.06^***^(0.05 – 0.06) | 0.077^***^(0.06 – 0.09) |
|  | **Missing** | 1198 (40%) | 338 (56.2%) |  |  |
| **Condom use in past year *^e^*** | **No** | 1261 (42.1%) | 166 (27.6%) | *Ref* |  |
|  | **Yes** | 945 (31.6%) | 310 (51.6%) | 2.16^***^(1.96 – 2.38) | – |
|  | **Missing** | 789 (26.3%) | 125 (20.8%) |  |  |
| **Condom frequency** | **Always** | 1281 (42.8%) | 181 (30.1%) | *Ref* |  |
|  | **Sometimes** | 783 (26.1%) | 221 (36.8%) | 1.81^***^(1.63 – 2.01) | 1.76^***^(1.49 – 2.10) |
|  | **Never** | 181 (6%) | 72 (12%) | 2.73^***^(2.31 – 3.23) | 4.47^***^ (3.42 – 5.84) |
|  | **Missing** | 750 (25%) | 127 (21.1%) |  |  |
| **Nights away from home** | **Zero** | 1568 (52.4%) | 270 (44.9%) | *Ref* |  |
|  | **< 1 week –< 1 month** | 603 (20.1%) | 133 (22.1%) | 1.21^***^(1.09 – 1.35) | 0.86(0.68 – 1.10) |
|  | **>1 month** | 822 (27.4%) | 198 (32.9%) | 1.53^***^(1.39 – 1.68) | 0.94(0.8 – 1.12) |
|  | **Missing** | 2 (0.1%) | 0 (0%) |  |  |

*^*^ - p<0.05, ^**^- p<0.01, ^***^- p<0.001*. n – number of participants, Und – undiagnosed, D –diagnosed, OR – odds ratio, aOR – adjusted odds ratio for clustering by community, CI – confidence interval, Ref – reference group, NA – not applicable (excluded men, only women were asked about number of children, women who refused to answer the question), ^a^ – univariate logistic regression, ^b^ – multivariable logistic regression. New cases are those that were newly diagnosed during BCPP, ^cd^ – the test performed in the study counts towards total tests thus all participants had at least 1 HIV test, ^de^ – partners are listed as HIV positive or negative based on the index partner’s knowledge of their status, ^f^ – “condom use” was excluded from multivariable analysis due to interactions with condom frequency.


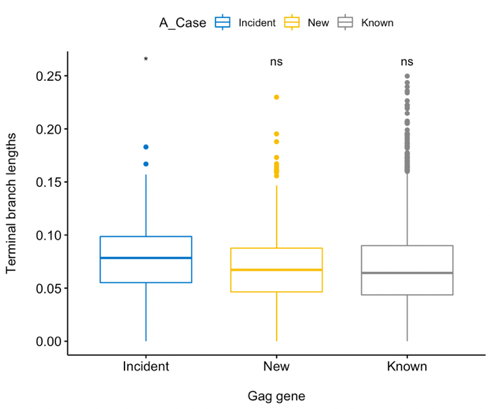

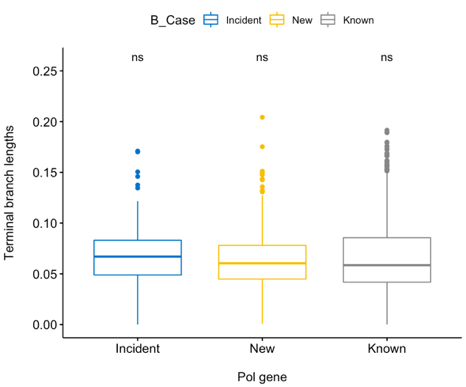

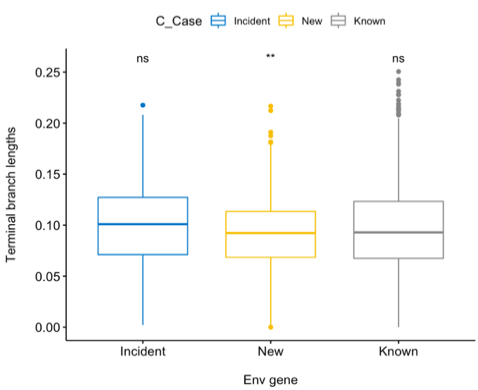


**Figure S1** Differences in terminal branch lengths for three gene regions: A. Gag, B. Polymerase and C. Envelope genes of the HIV-1C virus. Incident cases (blue), new cases (yellow) and known cases (grey) are shown in each plot. The Student’s t test was used to generate the *p*-values: ns -not significant, *^*^ - p < 0.05, ^**^- p < 0.01, ^***^- p < 0.001.* After Bonferroni’s correction, no statistical difference was observed in terminal branch lengths across the HIV genes.
